# Breeding success of a marine central place forager in the context of climate change: A modeling approach

**DOI:** 10.1371/journal.pone.0173797

**Published:** 2017-03-29

**Authors:** Lauriane Massardier-Galatà, Jennifer Morinay, Frédéric Bailleul, Eric Wajnberg, Christophe Guinet, Patrick Coquillard

**Affiliations:** 1 Université Côte d'Azur, Nice, France; 2 Université Côte d'Azur, INRA, CNRS, ISA, Valbonne-Sophia Antipolis, France; 3 Centre d’Etudes Biologiques de Chizé, CNRS, Villiers en Bois, France; University of California Santa Cruz, UNITED STATES

## Abstract

In response to climate warming, a southward shift in productive frontal systems serving as the main foraging sites for many top predator species is likely to occur in Subantarctic areas. Central place foragers, such as seabirds and pinnipeds, are thus likely to cope with an increase in the distance between foraging locations and their land-based breeding colonies. Understanding how central place foragers should modify their foraging behavior in response to changes in prey accessibility appears crucial. A spatially explicit individual-based simulation model (Marine Central Place Forager Simulator (MarCPFS)), including bio-energetic components, was built to evaluate effects of possible changes in prey resources accessibility on individual performances and breeding success. The study was calibrated on a particular example: the Antarctic fur seal (*Arctocephalus gazella*), which alternates between oceanic areas in which females feed and the land-based colony in which they suckle their young over a 120 days rearing period. Our model shows the importance of the distance covered to feed and prey aggregation which appeared to be key factors to which animals are highly sensitive. Memorization and learning abilities also appear to be essential breeding success traits. Females were found to be most successful for intermediate levels of prey aggregation and short distance to the resource, resulting in optimal female body length. Increased distance to resources due to climate warming should hinder pups’ growth and survival while female body length should increase.

## Introduction

Climate change scenarios supported by direct *in situ* observations have predicted an average sea surface temperature (SST) rise of 1–6°C by 2100 [1, 2], which could modify the distribution and habitat of many marine species [1–4]. Change in ocean heat content, mixed-layer depth, stratification and acidification have been noticed with direct consequences on ocean productivity [5, 6]. Polar oceans in both hemispheres are warming at a faster rate than elsewhere on earth [3] with direct effect on ocean circulation and location of frontal systems [7–9].

Large efforts are dedicated to quantify physical oceanographic changes. However, it is critical to assess the consequences of such changes on marine ecosystems and populations dynamic. In that context, it is crucial to understand and predict how changes in prey distribution and abundance, in response to environmental changes could impact the energy balance of marine top predators. To better understand population changes, it is of utmost importance to explore both the primary causes and underlying behavioral mechanisms impacting on individual fitness. Not to say how fitness is highly dependent on the ability of animals to feed in their environment.

A key issue is whether and how quickly predators can compensate for effects of environmental changes through short-term acclimatization or long-term evolutionary adaptation across generations and how phenotypic traits should vary to maximize individual fitness. Phenotypic traits are usually selected under the pressure of multiple environmental constraints [10]. This multiplicity can make it difficult to predict evolutionary trajectories under different prey distribution/abundance scenarios.

Such understanding might be critical to identify species at risk and for ecosystem managements. Pinnipeds and seabirds, which are central place foragers (*i*.*e*., they commute between their breeding (central) colony on land and foraging at sea), are constrained by particular distribution ranges of prey. Therefore, during breeding when they are restricted to a central place foraging strategy, pinnipeds such as otariids and non-flying seabirds should be more impacted by changes in prey distribution [8, 11–16]. Consequently, it is necessary to consider possible alterations in the structure of the prey fields in these environments. For instance, within the Southern Oceans, climatic scenarios predict a southward shift of the highly productive Polar Front [17], which separates Subantarctic waters from cold waters of Antarctica, with direct consequences on the foraging and breeding performances of king penguins *Aptenodytes patagonicus* [8] and other marine top predators [18, 19]. How central place foragers should modify their provisioning/foraging behavior to accommodate changes in prey accessibility in relation to their colony? According to the optimal foraging theory, foraging strategies are selected on the basis of maximization of the net energy intake rate [20] with an increase in load size with foraging trip duration for central place foragers [21].

Using computer simulations, we attempted to evaluate the potential changes in the breeding success of a central place forager in relation to foraging conditions that could be affected by climate changes (possible change in abundance or/and aggregation or/and accessibility of prey) and the intrinsic features of the parent (body length, foraging strategies and memorization abilities). To investigate separate and combined influence of these factors on breeding success, this study was calibrated on a particular central place forager model: the Antarctic fur seal (*Arctocephalus gazella* Peters, 1875) from the Kerguelen Islands which might be particularly vulnerable to a shift in the spatial distribution of their foraging ground [12, 13, 19]. Indeed, this species alternates, over its whole lactation period, between oceanic areas in which individuals feed for up to ten days, and short stays (one to three days) at the land-based colony to suckle their young [12, 13, 22–27]. Antarctic fur seals forage mainly on small mesopelagic fishes and can forage all around their central breeding place [22–28]. A large body of data is available for both females and pups. Females are solely responsible for rearing their pup (one per year) [22]. Depending on the environmental conditions, female body length may affect pup’s growth, pup mass gain and survival rate [16, 24, 25, 29].

We thus developed a spatially explicit bio-energetic simulator, using the individual-based modeling (IBM) technique [30–32], named MarCPFS for Marine Central Place Forager Simulator. This work mainly aimed at (i) verifying that outcomes were in accordance, at least qualitatively, with the optimal foraging theory predictions, (ii) understanding why and to what extent the possible environmental variations due to climate change could affect central place foragers’ survival and breeding success, (iii) testing the existence of optimal values for female body length related to the environmental parameters of our simulations (especially the distance to the resource).

## Materials and methods

### Study model

Female Antarctic fur seals forage in a complex marine environment, around the Kerguelen Islands (49°00′S, 70°00′E) [12–14, 22–26], in the vicinity of highly productive frontal systems: the Antarctic Polar Frontal Zone, which is delimited southward by the Polar Front and northward by the Subtropical Front [24, 33, 34]. Female fur seals feed almost exclusively at night at depth ranging from 20 to 60 m [24], when their prey resource (lantern fishes, *Myctophydae*) migrates towards the surface [23, 28].

Antarctic fur seals have one pup per year, which they rear from early December to late March [12, 22, 35]. This period, referred to as the “rearing period”. We can roughly distinguish several phases: (i) the prospective prenatal period (15 days), during which females move around the island, explore the environment and memorize the richest resources locations, (ii) the perinatal period (5 to 7 days): after giving birth, females remain ashore to feed their pup [12, 26, 35, 36], and (iii) the pup-rearing period (lactation period) covering the rest of the simulation (120 days) during which females alternate periods of two to nine days (about 5 days on average) at sea to fish and one to three days ashore to suckle the pup [12, 13, 22–27, 29, 37–40].

### Creation of the environmental maps

The first step consisted in creating artificial maps of resources in which females will move. The map, static over time, representing the environment is a 100 × 100 matrix. Each cell corresponds to a 10 × 10 km area and the island is set at the center of the map. According to Guinet *et al*. [15] females were found to forage up to 547 km away from the island, with a mean distance of 160 km. We therefore distributed the food resources around the islands within a 500 km radius circle. Female fur seals start foraging extensively beyond the Kerguelen plateau edge [15, 24 and 28]. Moreover, recent works have showed that there are few fishing attempts over the Kerguelen plateau compared to the pelagic zone, suggesting that the plateau is an unsuitable foraging area (40; M. Viviant unpublished data). The first step of the map creation involved computing the Euclidean distance of each cell to the island. Then, all cells more than 500 km away or within the 50 km wide Kerguelen plateau were set to zero abundance. The distance to resources (Dist) represents the inner limit of the highest possible abundance so that there is a gradient of probability (from 0.0 to 1.0) from the edge of the plateau to this limit (S1 Fig). We defined nine possible distances to resources (every 50 km between 100 and 500 km). Maps of prey were randomly drawn using the Gaussian field method (GaussRF function of the R software [41]; RandomField package [42]). To this end, for each map the parameter "scale" of GaussRF defined an aggregation level. We defined eight arbitrary levels of prey aggregation (Aggreg) from dispersed to highly aggregated (*i*.*e*. 0, 1, 2, 3, 4, 6, 8 and 10; see S1C Fig). For a defined quantity of resource, the lowest level of aggregation (level 0) represents more than 500 patches of about 1.5 cells. The highest level of aggregation (level 10) corresponds to an average of 10 patches of about 67.4 cells. Then, after normalization, a multiplicative coefficient was assigned to each map corresponding to its total abundance level. We defined four levels of abundance (Abund): low, intermediate, high and very high which correspond to average fishing success of approximately 90, 180, 270 and 360 g per hour, respectively.

### Simulation model

#### Entities, state variables and scales

The time step for the simulations was fixed to one hour and day/night alternation (16/8 hours) was taken into account. For each female, four main attributes were embedded in the data structure and were updated every hour: (1) location of the female (X_seal_, Y_seal_), (2) her energy (MJ), (3) her decision whether or not to fish and (4) her decision to return to the island or to continue foraging. The pup energy was updated similarly. Females and pups parameters are characterized by several state variables listed Table 1.

**Table 1:** Definition of model parameters.

Symbols	Units	Meaning
**Maps parameters**		
Aggreg		Aggregation level
Abund	g h^-1^	Abundance of prey level
AvgEnv		Mean abundance in the non-zero environment
th_t_		Threshold in prey abundance for which the probability of fishing is 0.5
Dist	km	Distance to the resource
D_island_	km	Distance separating the female from the island
d_max_	km	Maximal distance travelled
Rep_map_		Number of the current map replication
**Females parameters**		
Length	cm	Female body length
X_seal_		x coordinate of the female
Y_seal_		y coordinate of the female
E_seal_	MJ	Energy of the female
E_init_	MJ	Initial energy of the female
E_max_	MJ	Maximal energy of the female
E_wb_	MJ	Energy needed to return to the colony
E_mh_	MJ	Energy expenditure per hour
E_fish_	MJ	Energy gained by the female during fishing
Fmr_sea_	W kg^-1^	Metabolic rate at sea
Econtent_seal_	MJ kg^-1^	Energy content for a female
E_won_	MJ	Energy gained by the female during a trip
W_init_	kg	Initial mass of the female
W_seal_	kg	Mass of the female
SwimD	degrees	Swimming direction taken by the female
Mem		Type of memorization
ts	h	Time already spent at sea for this trip
t_suckling_	h	Time the female has spent to suckle the pup
Mta	days	Mean time ashore
T_max_	h	Maximum time allocable to a trip
t	h	Time step
Rep		Number of the current seal replication
**Pup parameters**		
E_pup_	MJ	Energy of the pup
Econtent_pup_	MJ	Energy content for a pup
W_pup_	kg	Mass of the pup
W_initpup_	kg	Initial mass of the pup
W_max_	kg	Maximal mass of the pup

#### Process overview and scheduling

**Fig 1:**
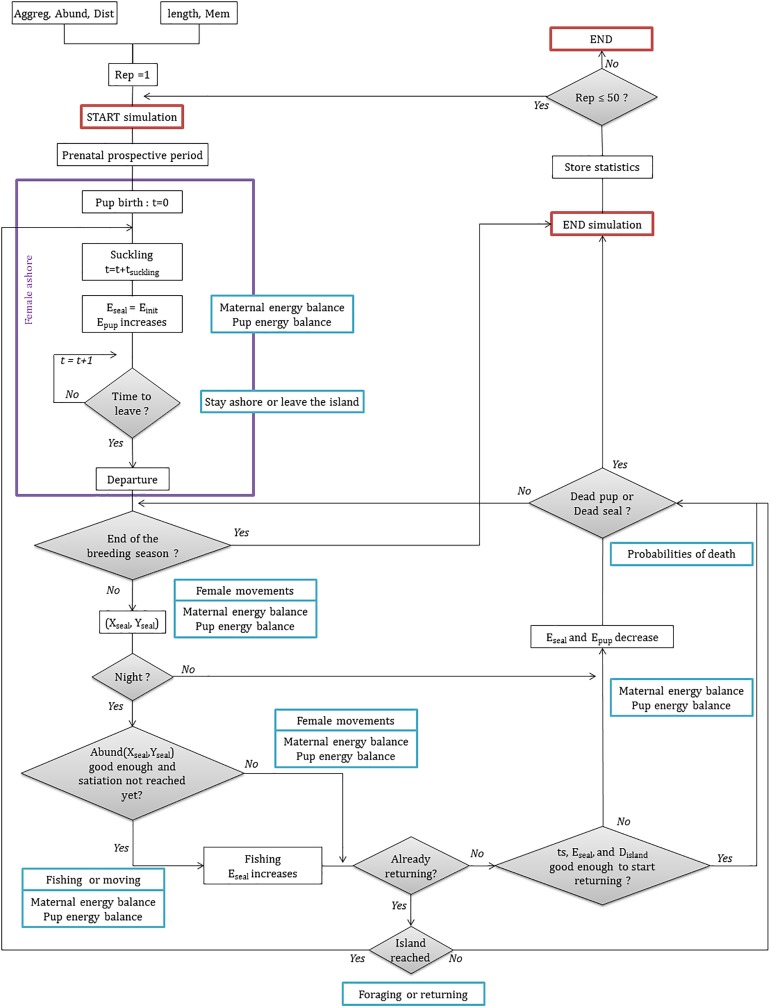
Model flow chart of the rearing period. Aggreg = aggregation level; Abund = prey abundance level; Dist = Distance to the resource; Length = female body length; Mem = type of memory. (X_seal_, Y_seal_) = location of the female in the current time. Abund (X_seal_,Y_seal_) = prey abundance at (X_seal_, Y_seal_) coordinate. Rep = number of the current seal replication. t = time step; t_suckling_ = time the female has spent to suckle the pup; ts = time the female has already spent at sea during the current trip; E_seal_ = current energy of the female seal; E_init_ = current initial energy of the female seal; E_pup_ = current energy of the pup; D_island_ = distance separating the female from the island (indirectly the time needed to go back to the island). The purple box corresponds to the periods the female is ashore. The blue boxes correspond to the submodels.

Several processes can be involved every hour, depending on the period and females’ location. MarCPFS processes are shown in the flow chart diagram (Fig 1). During the prospective prenatal period (15 days), the female explores the environment map and memorizes the richest resources locations. At the end of this period the pup is born and the time is set to zero. The simulation starts with both the female and its pup on the island. Every hour, both female's and pup's energetic status were updated (see Maternal energy balance and Pup energy balance sections below). Once at sea, the female travels directly to reach a memorized favorable feeding area or uses a correlated random walk (CRW) to forage within food-enriched zones or even a simple random walk when searching for new feeding areas (see Female movements section below). Every hour of the simulated period, the female has to make decisions. At night, the female is able to estimate the abundance of the map cell she is visiting and thus to decide either (1) to continue foraging or to return to the island (see Foraging or returning section below) or (2) to fish or to move (see Fishing or moving section below). When at colony, the female has to decide to stay ashore or to return at sea.

#### Submodels

**Maternal energy balance—**The energetic calculation depends on whether the female is at sea or ashore suckling the pup. When fishing, the energy gained by the female is proportional to the local abundance of prey. Fish intake is controlled by satiation, *i*.*e*. it cannot exceed 7% of the female’s energy [43] at the end of the former night and cannot exceed its absolute maximal energy E_max_ (; Table 2). Further details concerning energetic aspects and calculations are provided in S1A Text.

**Table 2:** Initial values of state variables.

Name		Value	Units	Source
**Map parameters**				
Aggregation levels	Aggreg	0, 1, 2, 3, 4, 6, 8 and 10		
Abundance of prey levels	Abund	90, 180, 270 and 360	g h^-1^	
Minimal value of abundance cell necessary to decide to fish		1.5 (≈ 55 g h^-1^)		
Distance to the resource	Dist	100, 150, 200, 250, 300, 350, 400, 450 and 500	km	
Map replication	Rep_map_	10		
**Female parameters**				
Female body length	Length	85,100, 115, 130 and 145	cm	
Type of memorization	Mem	0 and 1		
Breading period duration		4	months	[22]
Perinatal duration		5–7	days	[36]
Energy content for female	Econtent_seal_	10.59	MJ kg^-1^	[36]
Metabolic rate of the female at sea	FMR_sea_	6.09	W kg^-1^	[36]
Satiation rate of the female per feeding		7% of energy	MJ	[43]
Seal replication	Rep	50		
**Foraging trip**				
Maximal distance travelled	d_max_	500	km	[15, 24]
Swimming speed	v	2.11	m s^-1^	[27, 40]
Mean time ashore	Mta	2	days	[38]
Mean departure time		17	h	[26]
**Pup**				
Initial mass of the pup	W_initpup_	5.5	kg	[22]
Perinatal mass increase		1.5	kg	[44]
Energy content for the pup	Econtent_pup_	8.24	MJ kg^-1^	[22]
Maximal mass	W_max_	18	kg	
Mass lost per day		2.8% of energy	MJ	[26]

While at sea, the female loses energy corresponding to her mass-based metabolic rate at sea (6.09 W kg^-1^ h^-1^; [36]). To this value we added the fluid drag force generated by the displacement:

$$DragForce=\frac{1}{2}d\times S\times C\times v^{2}$$

Where *d* is the water density (1,034.7 g l^-1^), *S* the cross-sectional area (m^2^), *C* the drag coefficient and *v* the female speed (2.11 m s^-1^). The drag force was calculated for females ranging from 85 to 145 cm using a streamlined body shape (S1A Text). We therefore took into account the drag force generated by females when they move, using values ranging from 0.041 MJ m^-1^ (85 cm-long females) to 0.12 MJ m^-1^ (145 cm-long females).

**Pup energy balance—**The mass calculation of the pup depends on whether the female seal is at sea or ashore (S1B Text). The starving pup loses about 0.12% of his energy per hour [26]. After suckling, the energy of the pup increases, as described by Goldsworthy *et al*.[45].

**Stay ashore or leave the island**—Upon taking the decision to leave the land for a new trip, the female has to consider both the time required to reach the best patch she memorized during the previous trip and the current time. The female leaves her pup at a consistent time, taking into account the constraint of travelling to reach this location at night. Alternatively, females can stay one more night ashore and continue to suckle their pups.

**Female movements—**Previous theoretical and empirical studies demonstrated that foragers of a wide spectrum of species usually display movement patterns in accordance with a correlated random walk (CRW) [45, 46] and showed that grey seals movements were consistent with the correlated random walk for 48% of the seals tracked by satellite [47]. We compared autocorrelations between angles of simulated trajectories and trajectories obtained by satellite tracking (S2A Fig). In each case we found significant positive correlations between angles of successive time steps (0.302 for simulated trajectories and 0.218 for satellite tracking), but no correlation between angles recorded over wider intervals. Moreover, we implemented a pure random walk and preliminary simulations showed that mortality of both females and pups were so high that the results were incompatible with the persistence of populations (data not shown). We therefore retained the CRW as a reasonable assumption, using a constant swimming speed of 2.11 m s^-1^ [27] when the female moves horizontally. However, horizontal displacements were considered as negligible when the female was fishing (see Fishing or moving section below), resulting in a lower overall average speed within the foraging areas. A correlated random walk allows the female to estimate prey abundance in each of the cells visited and thus to determine whether there is a gradient between two contiguous visited cells. The female then uses this information to make a decision regarding which direction to go (S1C Text). At the very beginning of the simulation (*i*.*e*., the first hour of the prenatal period) the direction was chosen randomly, simulating the behavior of a naïve female.

**Fishing or moving**—The probability of fishing depends on various parameters. It was set to zero during the day, as lantern fish, their preferred prey, remain in deep waters and are out of reach for the fur seals. During the night, if a female was not at satiety, it was possible for her to fish, depending both on prey abundance and her energy status (Table 2). The satiation scales allometrically with body mass [48] and cannot exceed the maximal energy of the female which is equal to 1.07 times its mass at the end of the former night [43]. The probability of fishing was based on a few assumptions. First, a “poor zone” was defined as a zone where the abundance of prey is inferior to the mean abundance in the non-zero environment (AvgEnv). Second, the more abundant the prey, the higher is the probability for a female to fish. Third, we define the E_seal_ / E_min_ ratio where E_seal_ is the current content in energy of the female and E_min_ the limit energy levels below which it dies: the higher this ratio, the lower the probability of fishing in a poor area. This means that females in poor body condition will be more prone to fish on poor quality patches than others. Finally, below a minimal abundance of 1.5 in the cell visited (this value corresponding on average to 55 g of prey catchable in one hour) fishing is not profitable since the expenses due to fishing are higher than the gain. This minimal abundance will allow the female to collect at least 4×E_mh_ in one hour (where E_mh_ is the energy expenditure per hour and the constant 4 is the conversion unit of fish mass (g) into kcal; see Maternal energy balance section above). This energy intake rate is profitable as it compensates travel costs, and the non-fishing hours during the day. Finally, the probability to fish is:

$$P{\left(fishing\right)}_{i,t}=\frac{e^{2(prey\;abundance_{i}-th_{t})}}{1+e^{2(prey\;abundance_{i}-th_{t})}},$$

Where *prey abundance*_*i*_ is the abundance of prey at time t in the cell *i* and *th*_*t*_ is a threshold in prey abundance for which the probability of fishing is 0.5 (S1D Text).

**Foraging or returning**—The decision to return to the island depends on the current females' energy stock, the distance from the colony and the number of hours already spent at sea. E_wb_ is the energy needed for returning to the colony (*i*.*e*. the intrinsic energy expenditure of the female plus the energy needed to travel back to the island and the energy required for two days ashore to suckle the pup) divided by the maximal energy E_max_. E is the current energy E_seal_ divided by the maximal energy E_max_ and ts the time already spent at sea for this trip, divided by the maximum time T_max_ allocable to a trip. These variables allow computation of a probability to go back to the island (S1E Text).

**Probabilities of death***—*Females and pups have a probability to die which depends on their percentage of energy loss at time *t*, compared to a reference value. The lethal masses were estimated to be reached when females and pups were respectively weighting 70% and 56% of their reference masses, *i*.*e*., the mass at the end of the last suckling period [49]. The 56% of the initial mass at the beginning of a new fasting event was set according to field observations of pup mortality from starvation (C. Guinet, unpublished data) and 70% was set for females as they had already depleted their body store during the lactation period and which represented on average about 4.2 kg for an initial mass of 31.9 kg (*i*.*e*., 14% mass loss, [45]). To calculate these probabilities, we used Normal cumulative distributions: N(70, 0.02) for females and N(56, 0.02) for pups. The reference values were the energy after suckling for the pup, and the initial energy of the female (E_init_), respectively.

**Memorizing**—Many studies highlighted the importance of spatial memory for foraging efficiency, particularly among mammals [50, 51]. We hypothesized that the likelihood of heading in the same direction was a function of foraging success during the last trip [29]. In MarCPFS, females can remember the location of the richest cell they encountered. If later in the simulation they find a better cell, then that cell becomes the new reference. When they leave the island, females travel towards the reference cell with fidelity calculated on the basis of the energy gained during the previous trip divided by its duration (S1F Text).

#### Initialization

We defined several energetic variables specific to the females (for example the metabolic rate at sea [36]). These initial values are shown in Table 2. One of the two types of memory was assigned to each female. We considered the following two cases: no memorization at all (Mem0); memorization of the best patch location discovered during the prospective prenatal period and updating of this location throughout the pup-rearing period (Mem1).

The initial values of the foraging behavior such as the mean duration of the trip and the mean duration ashore to suckle the pup were obtained from the literature ([29, 38]; Table 2). We calculated the initial mass of the female from data collected on animals just before their departure from the island (C. Guinet, unpublished data). Equation (n = 147, *R*^2^ = 0.49) for initial female mass (kg) was:

$$W_{seal}=0.5246\times length-29.595$$

The female body length was fixed for each simulation (85, 100, 115, 130 or 145 cm). We finally defined several energetic variables specific to the pup (for example the pup's mass lost per day in percent ([24]; Table 2)). The initial mass of the pups was set to 5.5 kg [22].

#### Model output

**Simulation experiment—**For each simulation, we used a map where the prey abundance level (four levels; Abund), the aggregation level (eight levels; Aggreg) and the distance to the resource (nine distances; Dist) were fixed (Tables 1 and 2). Each map has been replicated 10 times (Rep_map_) to generate stochasticity in the repartition of prey (see Creation of environmental maps section). For each simulation, female fur seal can be initialized with a given body length (five lengths; Length) and one type of memorization (two types of memorization; Mem). Each simulation was repeated 50 times (Rep). In total, 1,440,000 simulations were performed.

**Output data***—*All results are expressed as means ± SD obtained on the basis of 10 replicated maps. At each hour, we recorded the location of the female (X_seal_, Y_seal_) and her energetic status. At the end of each simulation, we recorded whether the female and the pup survived and recorded their respective mass. For the foraging behavior, we considered only females that successfully completed their rearing period and we recorded the mean distance travelled per trip (MeanDTrip), the mean trip duration (MeanTTrip), the number of trips (NbrTrip) and the number of fishing events (NbrFishing) which was simply defined by the number of fishing decisions. Hence, when a female decided to fish (see Fishing or Moving section above), because of the time step we chose, the fishing activity of a single hour was recorded as a unique fishing event. We finally recorded the energy gained by fishing (E_fish_) during a fishing event.

**Female and pup survival**—Deaths of both female and pup were recorded in each simulation. To assess the overall reproductive success of the females, we calculated the average success of female-pup pairs (SP) in a given environment, for each of the 10 replicated maps, as follows: *SP* = (1 - D_f_) × (1 - D_p_), where D_f_ and D_p_ are the probabilities of death for females and pups, respectively.

**Cost/benefit ratio—**Biological systems could have, theoretically, an optimal (*i*.*e*. minimums) cost/benefit ratio (R) in the space of available energy [52]. We thus calculated for each female of each body length:

$$R=\frac{E}{SP}$$

with

$$E=\sum_{t=0}^{t_{r}}E_{t}$$

Where E_t_ is the energy spent during 1 hour and t_r_ = 2880 h is the total simulated duration of the rearing period, *i*.*e*. 120 days. Confidence interval of this minimum was obtained by the bootstrap method (1000 samples with replacement), fitting on each sample two different expressions and then calculating the 2.5 and 97.5 percentiles of the Min[*R*(*l*)] found. More details are available in S1G Text.

## Results

Both environmental and female parameters (Dist, Abund, Aggreg, Mem and Length) substantially modified the mortality and final mass of both the female and her pup. These parameters also influenced several characteristics of the female’s foraging behavior: MeanDTrip, MeanTTrip, NbrTrip and NbrFishing. Given the strong linear correlation (r = 0.99) between MeanDTrip and MeanTTrip, only the mean distance travelled was used.

### Abundance and aggregation of prey

**Fig 2:**
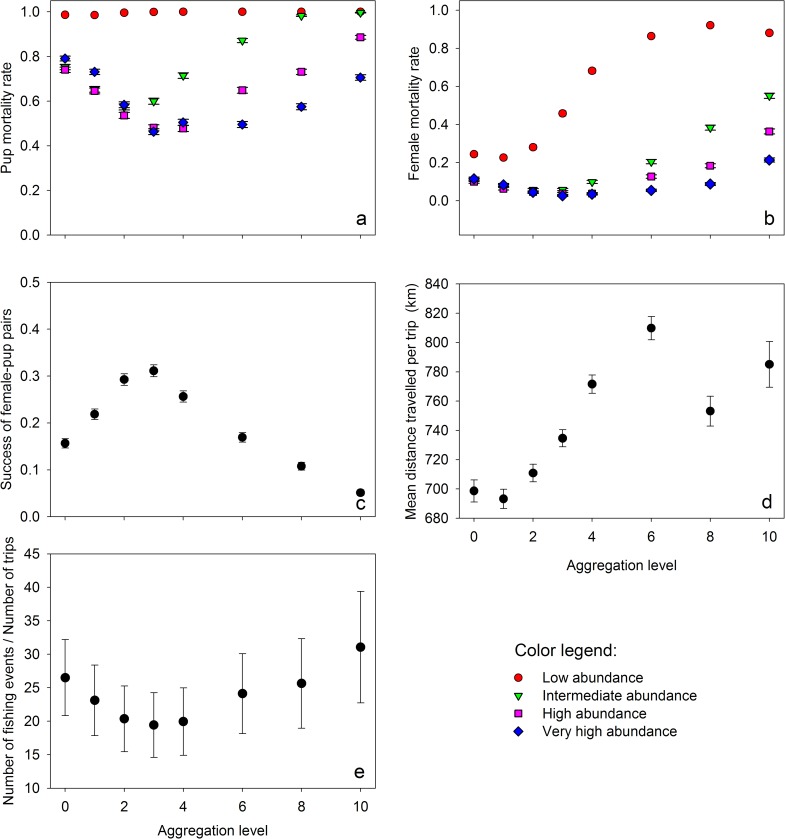
Effect of aggregation level of food resources. Effect (mean ± SD) of resource aggregation and abundance level on the probability of (a) pups and (b) females death: low (red circles), intermediate (green triangles), high (purple squares) and very high abundance (blue diamonds); n = 1350 in each case. (c) Mean (± SD) of the success of female-pup pairs (SP) as a function of the level of resource aggregation. (d) Effect (mean ± SD) of the aggregation level of the prey on the mean distance travelled by trips (MeanDTrip). (e) Effect (mean ± SD) of the aggregation level of the prey on the number of fishing events per trip (NbrFishing/NbrTrip). For both (d) and (e) we considered only females that successfully completed their rearing period.

The mortality rates of both females and pups increased with prey aggregation, except in conditions of low prey abundance, where the probability of pup death was always close to 1.0 (Fig 2A and 2B). Interestingly, the lowest pup mortality rates were observed with intermediate levels of food aggregation, in conditions in which prey abundance was sufficiently high. Female mortality varied most strongly when prey abundance was low. The lowest female mortalities were recorded with aggregation levels 2 and 3.

The success of the female–pup pairs (SP) was also highest for intermediate levels of prey aggregation (Fig 2C). In such conditions, the mortality rate was 14.5 ± 0.01% and 63.6 ± 0.01% for females and their pups, respectively. The corresponding mean final mass of the female across all body lengths was 36.2 ± 0.5 kg and 10.7 ± 0.2 kg for pups, including all tested distances to the resource. However, when retaining an average Dist of 150 km to feed [38, 39], and a female’s body length of 115 cm (this length corresponds to the mean female length at Kerguelen Island, C. Guinet, unpublished data.; [24]), simulated females weaned their pups at a mass of 11.0 ± 3.4 kg and reached a final mass of 32.7 ± 0.9 kg in moderate conditions of aggregation (level 3) and intermediate abundance of the prey.

Higher levels of prey aggregation were associated with a longer mean trip distance, up to aggregation levels 6 then MeanDTrip decreased (Fig 2D). Thus, an aggregation level 6 seems to be a critical threshold in terms of both the distance travelled and the time spent at sea per trip (five days: 119 ± 1.3 h). NbrFishing decreased monotonically with Aggreg, from 299.5 ± 1.0 (highly dispersed) to 233.9 ± 1.9 (highly aggregated). The NbrTrip was highest for an aggregation level 3 (14.5 ± 1.0). Finally, with aggregation levels up to 3, NbrFishing steadily decreased, as each successful dive provided a sufficient amount of food (Fig 2E).

### Distance to the resource

**Fig 3:**
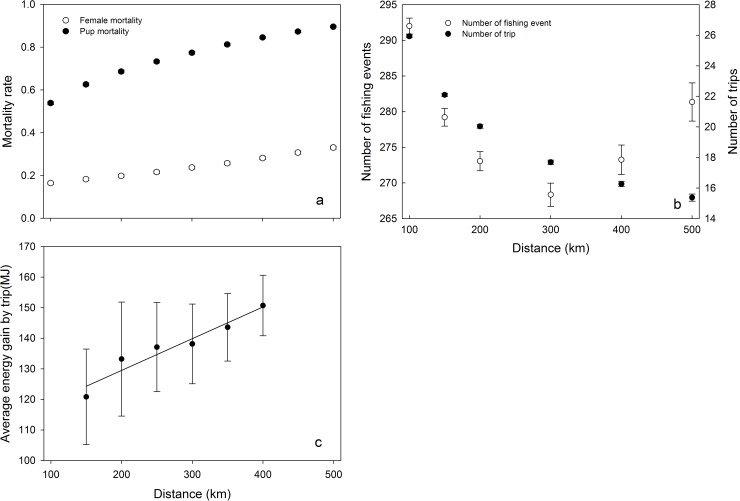
Effects of distance to the resource (Dist) on foraging behavior parameters. Effects (mean ± SD) of distance to the resource on (a) the probability of pups death (filled circles) and females death (open circles) (n = 4800 in each case), (b) the fishing events number (NbrFishing, open circles) and the trips number (NbrTrip, filled circles) (n = 4800) and (c) the average energy gained by females by trip (n = 1598). Parameters used: Aggreg = 3, Abund = intermediate level, female body length = 115 cm and type of memorization = Mem1. The black line represents the linear regression with R^2^ = 0.93. For both (b) and (c) only females that successfully breed their pup were considered.

Distance to the resource had a strong impact on mortality (Fig 3A) and final mass (data not shown) of both the female and her pup. Greater distances were associated with higher mortality (Fig 3A). In extreme cases, in which the distance to the resource was particularly large, the mean final mass of the pup was below its initial mass.

The MeanDTrip over the rearing period was proportional to Dist (from 568.8 ± 3.5 km to 1,010 ± 8.5 km, Dist ranging from 100 to 500 km; R^2^ = 0.94). Similarly, the time spent at sea (MeanTTrip) increased with mean distance travelled (from 85.7 ± 0.6 h to 150.7 ± 1.3 h). However, the NbrTrip was reduced when Dist increased (Fig 3B). The NbrFishing also varied with Dist, decreasing to about 300 km and then increasing again.

In Fig 3C, we fixed other parameters (Aggreg = 3, Abund = intermediate level, female body length = 115 cm and type of memorization = Mem1) and considered only females that successfully breed their pup. The E_fish_ by trip increased with the distance to the resource. However, the sum over the breeding period of the weight gain by the pups decreased with Dist (ranging from 31.2 ± 7.2 kg to 19.1 ± 2.3 kg).

### Memorization

**Fig 4:**
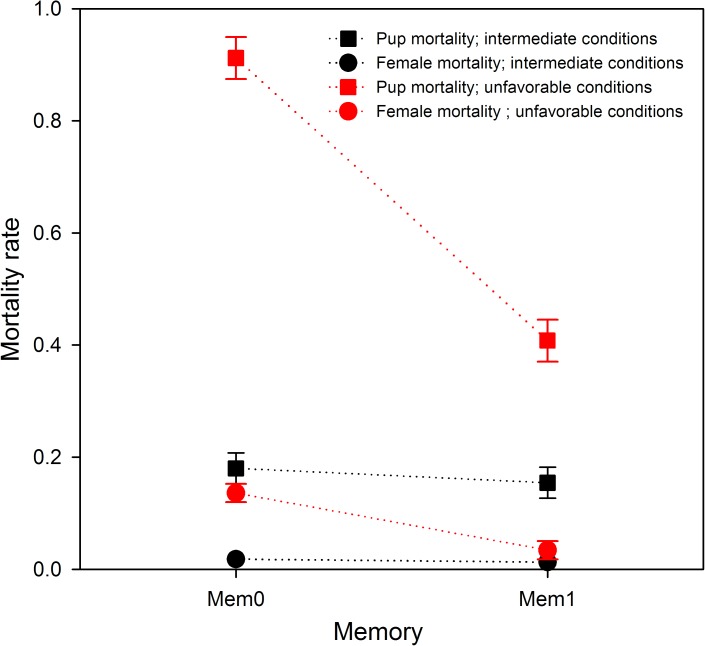
Effect of the memorization on mortality rates. Effect (mean ± SD) of memorization on the probability of pup death (square symbols) and on the probability of female death (circle symbols). Simulations involved females of 115 cm body length only. Intermediate conditions (black color): prey aggregation level 3, distance to the resource = 150 km, intermediate prey abundance. Unfavorable conditions (red color): prey aggregation level 1, distance to the resource = 300 km, intermediate prey abundance. Simulation performed using Mem0 (no memory) and Mem1 (memorization of location of the best patches).

**Fig 5:**
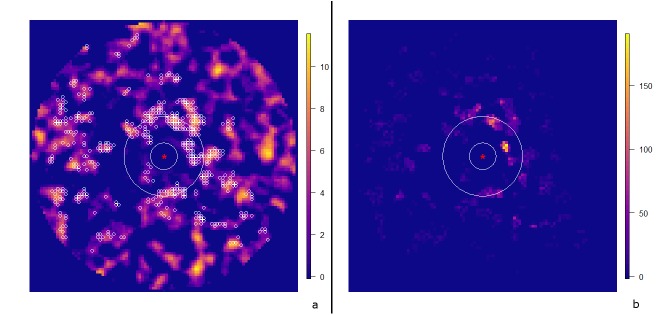
Distribution and frequency of fishing events. Simulation results for 115 cm female body length in an environment roughly corresponding to that of the Kerguelen archipelago (intermediate abundance = 180 g of catchable prey per hour, Aggreg = 3 and Dist = 150 km). The red star identifies the location of the islands; the two white circles define the limits of the island plateau and the distance (Dist) to the resource, respectively. (a) Localization of the fishing events of 10 females over the rearing period (small white circles) on the map of resource density. (b) Location and frequency of the fishing events of 50 females over the rearing period in the same environment. One can see that the most frequently fished cells (b) mainly correspond to the richest ones (a). Conversely, a lot of poor cells have been visited in which animals have been fishing. However, these cells show low fishing frequencies indicating that animals are likely to spend less time in poor-quality zones.

The ability to memorize significantly affected the mortality rates of both females and pups. When retaining parameters according to the literature (body length of 115 cm; Dist = 150 km), and under quite favorable conditions (Aggreg = 3 and Abund = intermediate level), the memorization ability had no appreciable effect on the mortality of females or pups (Fig 4). Conversely, in unfavorable conditions (female body length of 115 cm; Dist = 300 km; Aggreg = 1; Abund = intermediate level) both females and pups took advantage from the memory ability (Fig 4). Finally, for a given value of Dist, among the richest zones, the most frequently fished are the ones closer to the island (Fig 5).

### Female body length

**Fig 6:**
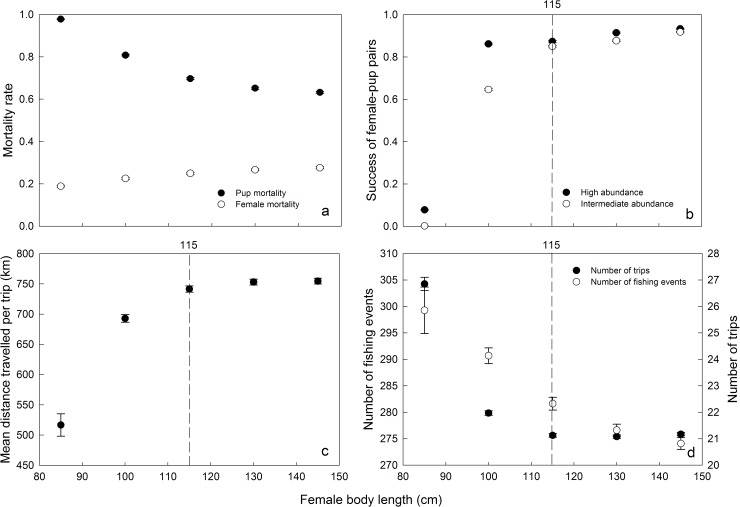
Effects of female body length on survival and foraging behavior. Effect (mean ± SD) of female body length on (a) the mortality rates of pups (filled circles) and females (open circles) (n = 8640 in each case), (b) the success of the pair mother/pup (SP) (intermediate conditions: Aggreg = 3, Dist = 150 km, high prey abundance (filled circles) and intermediate prey abundance (open circles)) (n = 8640 in each case), (c) the distance travelled per trip (MeanDTrip), and (d) the fishing events number (NbrFishing, open circles) and the trips number (NbrTrip, filled circles).

Body length had a positive, although weak, effect on female mortality and a stronger negative effect on that of pups (Fig 6A). We calculated the success of female-pup pairs using Aggreg = 3 and Dist = 150 km, in two conditions of abundance (intermediate and high). In such conditions, females with a body length of 115 cm or more were the most successful (*SP* > 0.8; Fig 6B). On average, the longest females (≥ 115 cm) performed the longest trips (Fig 6C and 6D). Conversely, NbrFishing and the NbrTrip both decreased with increasing female body length. Thus, longer females exploited distant resources more efficiently than shorter ones.

### Identifying optimal body length

**Fig 7:**
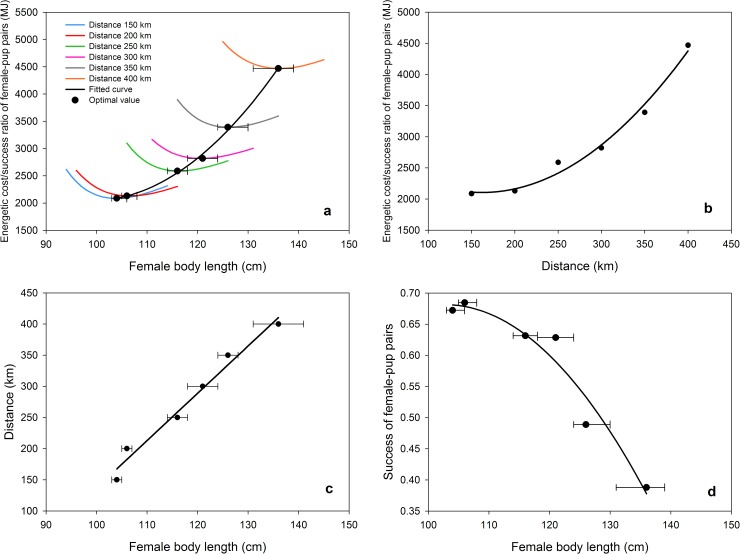
Identifying optimal body length. (a) (Energetic cost) / (success of female-pup pairs) ratio (*R* = *E*/*SP*) as a function of female body length, for increasing distances from colony to the resource areas. Each color represents the distance to the resource (Dist) (blue: 150 km, red: 200 km, green: 250 km, pink: 300 km, grey: 350 km, orange: 400 km). Horizontal line indicates the 95% confidence intervals obtained by the bootstrap method (1000 samples with replacements). The regression curve *R* = 1.792*Length*^2^-355.4*Length*+19670 (R^2^ = 0.99) is only indicative. To clarify the graph, the representation of each relation was limited to values close to the optimum. (b) Optimal E/SP ratios (MJ) as a function of Dist. (c) Distance–optimal female body length relationship. (d) SP-Optimal female body length relationship. Parameters used: Aggreg = 3, Abund = intermediate level and type of memorization = Mem1.

Calculation of the (energetic cost) / (success of female-pup pairs) ratio (E/SP) revealed that, for each distance, there exist a minimal value corresponding to an optimal female body length. These optimal body lengths increased with the distance to the resource (Fig 7A). The E/SP ratio looks like a quadratic relationship with the distance the females have to cover (Fig 7B). When considering the optimal female body length as a function of the distance to be covered we find a positive linear relationship (Fig 7C). Outcomes showed that the success of the female-pup pairs (SP) should decrease in a quadratic relation with the female body length and consequently with the average distance that must be covered per trip (Fig 7D).

## Discussion

MarCPFS has several limitations since it considers variations in the horizontal plane only, whereas real females forage in a three-dimensional space. Resources considered in the model are static over time and not distributed in accordance with oceanographic features. The overestimation of final weights can be explained by the uncertainties about real prey abundance and aggregation levels, or the unknown effect of competition. Furthermore, diseases and predation are certainly factors of great importance in the real system. However, the consistency between the emerging results of our model and available observations, and the absence of neither abrupt nor abnormal bifurcation or excessive sensitivity to parameters, suggests that this simulator behaves in a satisfactory manner (Table 3).

**Table 3:** Elements for model validation.

	Sources		Results (simulations)
Pup's survival rate (%)	77.6 ± 20.1	[53]	86.2 ± 2.21
Female final weights (kg)	30.4 ± 0.7	[14]	32.67 ± 0.92
Pup final weights (kg)	10.5 ± 0.5	[14]	11.04 ± 3.42
Female length (cm)	114.8 ± 0.7	[14]	Optimal body length ≈ 115 cm
	No effect in favorable years	[14, 26, 55]	No effect if ≥ 115cm in intermediate to highly favorable conditions (Fig 5B)
Increasing distance of resource	Pup's rate mass gain decreases.	[26]	Pup's rate mass gain decreases.
	Time spent diving decrease.	[38]	Time spent diving decreases to a certain distance (depending on the female body length)

Simulation parameters: Dist = 150 km; female body length = 115 cm; Aggreg = 3; Abund = intermediate level; type of memory = Mem1.

### Model validation

To validate MarCPFS outputs, we compared model outputs to the real pup's survival rate from Reid and Forcada [53] and the data on final mass of female and pup from Lea *et al*. [14]. When using parameters reported by field measurements (Dist = 150 km, female body length = 115 cm [17]), moderate aggregation (level 3) and intermediate abundance (180 g h^-1^), we found final weights corresponding to the actual final weights of females and pups (32.7 ± 0.9 kg, 11.0 ± 3.4 kg for the simulated animals, versus 30.4 ± 0.7 kg, 10.5 ± 0.5 kg for females and pups, respectively ([14]; see Table 3). Under such conditions, simulations yielded a survival rate for pups of 86.2 ± 2.2%, which is comparable with field observations: 77.6 ± 20.1% ([53]; Table 3). Among all the possible combinations of initial parameters, only this particular combination provided results that replicate field values. We thus hypothesize that these conditions constitute an appropriate approximation of the real spatial distribution of prey. We also used some validation elements of foraging behavior of the female described by Boyd [38] (Table 3). The comparison of our results with those of Langton *et al*. [54] about the guillemot showed the convergence of the two systems with a special emphasis on the effects of distance and abundance of the resource on the foraging strategies. This constitutes a partial cross validation of the models in the context of marine central place foraging.

### Environmental parameters

All environmental variables (Dist, Abund and Aggreg) had direct effects on the foraging behavior of females, their mortality rate and final weight. In particular, the response of breeding success to prey spatial distribution parameters was not monotonous, exhibiting a maximum at the intermediate level of aggregation of prey (minimum of pup mortality). The effect of prey aggregation on the reproductive success can be partly explained by female behavioral changes when searching for food (see below). Several environmental conditions (extreme dispersal (Aggreg levels 0 and 1) and/or extreme aggregation of resources (Aggreg levels 8 and 10) and/or low abundance (90 g h^-1^)) are clearly unusual or exceptional, as they do not seem to be able to sustain sufficient successful breeding levels (Fig 2A and 2B). The strong negative effect of the distance to the resource on survival rate (Fig 3A) was expected and can be explained by the overall energy expenditure increasing with the distance, as already reported by Staniland *et al*. [55].

### At sea: The foraging behavior

This study highlights the importance of a sufficient level of resource aggregation to sustain populations, as already suggested by field studies (*e*.*g*., [15]). Aggreg and Abund strongly affected the number of fishing events per trip (Fig 2D and 2E). In particular, an increase in the aggregation level led to changes in the time spent searching for resource patches of sufficient quality and the mean distance travelled per trip. The curve for the number of fishing events per trip was convex in shape, with a minimum value of 19.4 ± 4.7 fishing events per trip (Aggreg = 3; Fig 2D and 2E). Indeed, for moderately aggregated resources, each fishing event provided a larger amount of energy compared to conditions of dispersed resources. Beyond this threshold value, the aggregation of prey requires animals to spend an even longer time looking for resources patches of sufficiently high quality.

The minimum number of fishing events over the rearing period was obtained for a distance of 300 km (Fig 3B). Beyond this distance, females must increase their fishing efforts to counterbalance the cost of covering such distances. As a consequence, females in poor body condition and unable to quickly recover a sufficient corporal energy are less likely to return to suckle their pup and more likely to abandon them to insure their own survival.

Memorizing location of the richest patches provided an advantage under harsh conditions (Fig 4). This suggests that memorization might be a trait of great importance in most populations of marine predators repeatedly facing such conditions. Indeed, Nable-Nielsen *et al*. [46] showed that simulations integrating animals’ ability to remember the profitability and location of previously visited areas were “able to reproduce the movement patterns that resembled those of real porpoises […] and to maximize their food intake.” For terrestrial mammalians, Merkle *et al*. [56] experiments produced similar outcomes and conclusions.

Finally, an ability to memorize the location of the best patches of resources enabled adults to decrease both the mean distance and duration of each trip and the number of fishing events (Fig 5).

Qualitatively, our results are consistent with the optimal foraging theory. According to this theory, animals are likely to spend less time in poor-quality patches and more time in high-quality patches of resources [17, 57]. Our results suggest that females finding cells, or patches of cells, with a low energy content tend to move away rapidly and search for better foraging locations (Fig 5). However, it could be argued that this finding reflects the presence, in the design of the simulator, of components of such behavior (*e*.*g*., correlated random walk movement). We also parameterized the decision making process for diving with the body condition of the animal, because animals lacking energy have to eat, even when confronted with poor patches of resources (see the Fishing or moving section above). These last two features of the model may, at least partly, cancel each other out, and we consider the observed consistency with the optimal foraging theory to be rather an emergent property of the model than a built-in mechanism (Fig 5).

As for central place foragers, the optimal foraging theory predicts that the load size should increase with the distance travelled from the colony to reach the feeding area [21]. Additionally, regular food provisioning of pup was found to be a critical factor underlying pup growth performance in Subantarctic areas [44] and constitutes a strong constraint over this type of population. Indeed, our results show that the average energy gained by trip increases with Dist (Fig 3C). On the other hand, the female energy expenditure also increases with Dist. As a consequence, longer distance greatly decreased the growth performances of the pups, which have to fast for prolonged periods of time. These results are consistent with previous findings for fur seals from the Kerguelen Islands showing that the rate of pup mass gain decreases with foraging-trip duration [26]. Lastly, carrying heavy loads affects the energy budget of central place foragers, increases travel costs and predation risks [58] and may harden the task of breeding pups.

### Optimal phenotype

Female body length was found to have an effect on the probability of successfully rearing a pup (Fig 6A). The shortest females were the least successful mothers, in accordance with Beauplet *et al*. [59]. Globally, when considering all conditions simulated, small females (≤110 cm long) making large numbers of short trips displayed the lowest levels of breeding success (89% pup mortality), whereas larger females (≥ 130 cm) making a lower number of trips were widely more successful (63% pup mortality) (Fig 6C and 6D). Bigger females can store larger amounts of body reserves [60] and are able to exploit efficiently distant food resources [61]. As already shown, they must increase the number of fishing events to compensate for energy loss resulting from both the distance covered and the drag force they undergo (Figs 3B, 6C and 6D). However, their mortality rates were higher than for small females mainly due to their larger metabolic requirements and higher displacement costs. On the contrary, when considering intermediate/favorable conditions only, there was no appreciable difference in the breeding success (SP) when females were ≥ 115 cm in length (Fig 6B).

The positive relationship between the optimal E/SP ratio and female body length (Fig 7A) can be interpreted as a positive correlation between the amount of lipids animals can store and their body length, an allometric scaling relating body length to mass. As already shown by Beauplet and Guinet [60], the longest females are more efficient at exploiting distant food resources. On Amsterdam island (1,500 km north of the Kerguelen islands), where female Subantarctic fur seals (*A*. *tropicalis*) have to perform long foraging trips, body length has been shown to be a critical factor for foraging performance [44] and survival [60, 62]. Furthermore, a population from South Georgia of *A*. *gazella* has also been shown to display a similar tendency, associated with decreased availability of Antarctic krill, due to the positive values of the Southern Annular Mode over the last three decades [63]. At Kerguelen Island depending on the foraging conditions female size were found to have a significant effect on pup growth under poor conditions, while no effect of female size on pup growth could be detected under favorable conditions [14, 26, 64]. However, our results are consistent with these observations and show that, under intermediate conditions (particularly Dist = 150 km), the success of female-pup pairs was roughly similar for females’ length ranging between 115 and 145 cm (Fig 6B) and it follows that “larger is not always better” [60].

The relationship between the E/SP ratio and the average distance to fishing areas is a positive quadratic one (Fig 7B). This is not very surprising since the drag force females undergo when they move, and the energy needed for it to be counter-balanced, is a function of the cross-sectional area (m^2^) of the animal. On the other hand, even if the animals can dynamically adopt optimal body lengths, SP evolves in a negative quadratic way (Fig 7D), which means that females have more and more difficulties to raise their pup with increasing distances. When looking at the optimal body length in function of Dist, we found a linear relationship (Fig 7C). L being the average length of the animals, we obtain:

$$L=\frac{k_{2}}{k_{1}}(Dist-D_{0})+L_{0}$$

where D_0_ is the initial value of distance, L_0_ the initial value of female body length, *k*_1_ and *k*_2_ the speeds at which the fishing areas and the female body length can change respectively (S2 Text). Simulations gave similar outcomes (Fig 7C), with a slope of 0.128 corresponding to *k*_2_/*k*_1_. For instance, using a constant speed (3 km year^-1^) for the shift of the polar front, female body length should increase on average by 0.38 cm year^-1^ for maintaining the best E/SP ratio. Under a pessimistic scenario in which females fail to modify quickly enough their phenotype (*i*.*e*. *k*_1_< 0.38 cm year^-1^), while the population has to go farther and farther at sea, the pups will have to fast for longer periods and will be less and less well-fed. In the end, even if the females could adjust their body length from generation to generation, their individual fitness should not evolve in a similar way. To that regards, Beauplet *et al*. [65] found that both survival of Subantarctic fur seal pups and post-weaning pup survival were strongly correlated with growth rate during the lactation period. In addition, we could fear that difficulties undergone within the lactation period should hinder pup’s growth resulting into smaller animals, since such a malnourishment leads to a growth delay that cannot be later fully compensated for in mammals [66, 67]. Lastly, in most dramatic cases, food distress could possibly lead to the population extinction.

## Conclusion

Our model shows the importance of the distance covered to feed and prey aggregation which are key factors to which animals are highly sensitive. Indeed, a simultaneous increase in distance and aggregation would be of dramatic consequences on the survival of central place forager populations. Such results should be considered within the framework of the environmental modifications likely to be triggered by climate change, as predicted by climatologists (*i*.*e*. a southward shift of the highly productive Polar Front). Several studies have already revealed relationships between oceanographic conditions and the foraging success of marine predators [12–15, 18, 19, 68, 69]. Environmental features and oceanographic cues including temperature, temperature gradients and water turbidity, may be indicative of phytoplankton concentration [70]. These cues may provide marine predators with elements allowing them to identify the most favorable locations. Thus, in view of the predicted climate change that could lead to critical conditions, the adaptive capacities of central place foragers is of major importance regarding their survival and conservation. Modelling outcomes suggested that only the largest females would be able to rear their newborns successfully if the distance to the resource increases. On the contrary, young (short) females which reach their definitive body length at about 7 years old would be disadvantaged in addition to their inexperience in the foraging activity [13, 22, 26, 28, 43]. Consequently, their contribution to successive generations could be increasingly limited in the course of time.

Body length appears to be a good example of an integrative trait [71–73]. It is known to be highly heritable in most animal species [74] and thus most likely under strong natural selection in the wild. Hence, we can hypothesize that analogous changes in the environment would lead to a gradual selection of larger individuals, although female length may not evolve quickly enough to compensate for the rate of change in the distance to resource parameter. This will depend on the adequacy of velocities of the southward shift of the main oceanographic frontal structures and evolution of the populations with regards to global warming.

Future studies should also consider the effect of the vertical accessibility and structuring of the prey on the reproductive success of females, as a function of their length. In this line, the work of Benoit-Bird [75] on several top predators (including the Northern fur seal (*Callorhinus ursinus*)) revealed that the “fine scale distribution of prey is critical to how predators perceive the suitability of their food supply and the mechanisms they use to exploit it”. In particular, the quality of the patches of juvenile Pollock on which Northern fur seals feed, their structuring into clusters and inter-patch distance, were found to play major roles. In order to obtain more detailed and accurate results, we have already engaged in coupling MarCPFS with a physical-biogeochemical model of the ocean dynamics (including the micronekton dynamics: SEAPODYM, [76]). This coupling, after modifications and new calibration of our model, should allow us to verify that some of the prominent results we presented can be, to some extent, generalized to other central place foragers such as seabirds.

Simulation code is available on request.

Synthetic simulation results are available on the MarCPFS DataBase: http://www2.sophia.inra.fr/MarCPFS_Database.

## Supporting information

S1 Text Details about submodels. (PDF)

S2 Text Details about the linear relationship between distance to the resource and female body length. (PDF)

S1 Fig Details about creation of environmental maps. (PDF)

S2 Fig Comparison between real tracking and tested movements in the simulator. (PDF)

S1 Dataset Data used to calculate the drag force. (TXT)

S2 Dataset Probability matrices of returning to the island (S1A Fig). (TXT)

S3 Dataset Seal length and mass data. (TXT)

S4 Dataset Energy spent used to calculate cost/benefit ratio. (TXT)

S5 Dataset Simulation results for distances ranging from 100 to 250 km. (ZIP)

S6 Dataset Simulation results for distances ranging from 300 to 500 km. (ZIP)

S7 Dataset Energy gained by trip. (TXT)

S8 Dataset Weight gained by the pup. (ZIP)

S9 Dataset Matrices used to build the Fig 5. (ZIP)
