# Supplementary material for: Breeding success of a marine central place forager in the context of climate change: A modeling approach
Source: PLoS One. 2017 Mar 29;12(3):e0173797. doi: 10.1371/journal.pone.0173797 (PMC5371308; doi:10.1371/journal.pone.0173797)
Supplement: S1 Text — (PDF) [file pone.0173797.s001.pdf]

S1 Text. Details of submodels

A. Maternal energy balance

- The initial energy (E_{init}) of the female was calculated as:

$$E_{init} = W_{init} \times Econtent_{seal}$$

where W_{init} is the initial mass of the female and $Econtent_{seal}$= 10.59 MJ kg-1 is the energy content for a female [1].

- The calculation of the body-mass during simulation was made using the same equation. The maximal energy of the female (E_{max}) is a theoretical upper limit of the energy gain for a female and was calculated as:

$$E_{max} = 1.20 \times W_{init} \times Econtent_{seal}$$

where W_{init} is the initial mass of the female and E_{max} was estimated from Arnould & Boyd [1].

- The energy gain by fishing (E_{fish}) was:

$$E_{fish} = 4 \times Abund(X_{seal}, Y_{seal}) \times \frac{E_{mh}}{1.5}$$

where 1.5 represents a minimal abundance of prey to ensure the energetic stasis of the animal, Abund (X_{seal},Y_{seal}) is the abundance value of the cell visited by the female at (X_{seal},Y_{seal}) coordinates. The constant 4 is the conversion unit of fish mass (g) into kcal.

- The minimum energy needed to go back to the island (E_{wb}) and to take the decision as:

$$E_{wb} = \left(time_{to\_island} \times E_{mh}\right) + \left(2 \times 1.002 \times Econtent_{seal}\right) + E_{init}$$

Where $time_{to\_island}$ is the time required to return on the island and 1.002 is a constant estimated for female mass loss during an attendance bout [2].

- The energy gained by the female during a trip (E_{won}) was defined as:

$$E_{won} = E_{seal} - E_{init}$$

- We defined the mass loss by the female ashore in two days as:

$$MJ\_lost\_island\_2days = 0.03 \times W_{seal} \times Econtent_{seal} \times 2$$

The female fur seal loses 3% of her mass ashore in two days [3]

- The female fur seal energy after suckling is:

$$E_{seal}\left(after\ suckling\right) = E_{seal} - E_{won} = E_{init}$$

- The energy expenditure of the female per hour (E_{mh}) was defined by:

$$E_{mh} = W_{seal} \times \frac{FMR_{sea}}{278}$$

where W_{seal} is the mass of the female, FMR_{sea} = 6.09 W kg^{-1} h^{-1} [1] the female metabolic rate at sea and 278 is the conversion factor from Watts to MJ h^{-1}.

- The hourly variation in energy of the female fur seal when at sea ($E_{seal}(t)$) was calculated as:

$$E_{seal}(t) = E_{seal}(t-1) \times [1 - \frac{FMR_{sea}}{278} \times \frac{1}{Econtent_{seal}}] + fishing \times E_{fish} - DragForce$$

If the female decides to fish, fishing = 1 and if not fishing =0 and DragForce is the fluid drag force generated by displacement.

Note: Thermoregulatory process could have been included in our model, depending on the volume/surface area of the simulated animals and of sea temperature at various depths. Indeed, the surface area increasing proportionally less rapidly with respect to the length, a thermoregulation process would add a disadvantage to small females. However, it would have been really difficult to take into account all the energetic parameters in their finest details and would have make sense in the context of a detailed simulation of the process of diving with a small time step (< 1 hour). In addition, considering the entire breeding season (4 months) would have strongly increased the complexity of the model and the computing time.
Finally, we considered that adding a thermoregulatory term would actually lead to qualitatively identical results with no significant modifications in results analysis and conclusion.

- While at sea, the female loses energy corresponding to her mass-based metabolic rate at sea (6.09 W kg^{-1} h^{-1}; [1]). To this value we added the fluid drag force generated by displacement:

$$DragForce = \frac{1}{2} d \times S \times C \times v^2$$

where *d* is the water density (1,034.7 g l^{-1}), *S* the cross-sectional area (m²), *C* the drag coefficient and *v* the female speed (2.11 m s^{-1}). The cross-section was calculated for females ranging from 85 to 145 cm using the following linear equation obtained by regression on collected data (C. Guinet, unpublished data):

$$c = 0.637 length - 0.025$$

where *c* is the maximum circumference and length is the female body length. The drag coefficient *C* was calculated using the ratio

$\lambda = \frac{length}{diameter}$ of the animal and considering that the females have a streamlined body shape.

We did not introduce a dynamical variation of the drag force for two reasons. First, positive and negative variations of weight (opposite values of going and returning phases of the trips) should counter-balance each other and result in an average value. Second, females make short trips only (5 days on average) and simulations revealed a weight variation during a trip of about 12% which induces a variation in diameter of 2 cm and thus a 3.4 10^{-4} MJ h^{-1} (*i.e.* 4.6 ‰) variation of the drag force. In such conditions we considered the impact on the cross section of the animal as negligible in terms of drag force.

B. Pup energy balance

- The mass calculation of the pup (W_{pup}) was defined by:

$$W_{pup} = \frac{E_{pup}}{Econtent_{pup}}$$

Where E_{pup} is the energy of the pup and $Econtent_{pup}$ = 8.24 MJ $kg^{-1}$is the energy content for a pup [4].

- When the female is at sea, the pup loses 2.8% of its mass per day [1, 3] and the hourly energy expenditure of the pup (E_{pup}) is:

$$E_{pup}(t) = E_{pup}(t-1) \times (1 - 0.028/24)$$

- When the female is ashore, we considered two cases: if the female does not have enough energy to feed the pup, it loses 2.8% of its mass per day and if the female has enough energy to feed the pup, the variation of its mass is [2]:

$$W_{pup}(t) = W_{pup}(t-1) + \left(0.9 \times \frac{E_{won}}{Econtent_{seal}} - (1.002d \times 0.9) \right)$$

Note: Guinet *et al.* [3] showed that "the maternal allocation does not differ according to the sex of the pup". According to Goldswothy *et al.* [2], we found no significant difference between females and male pups (Wilcoxon rank test P-value = 0.28; mean of pup mass-gain (PMG) for females 2.13 kg, n=4 and for males 2.57 kg, n=9). Hence, we considered in the model that there should not be distinction.

C. Female movements

A correlated random walk allowed the female to estimate prey abundance in each of the cells visited and, thus, to determine whether there was a gradient between two contiguous visited cells. The female then uses this information to make a decision regarding the direction in which to head. Thus, we defined a swimming direction (SwimD) which obeys these expressions:

$$SwimD = SwimD \pm N(\mu_1, \sigma_1)\frac{180}{\pi},$$

where $$\mu_1 = -\arctan(10 \times (\Delta Ab + 1) + \frac{\pi}{2}$$

and $$\sigma_1 = \frac{1}{3}\left(\arctan(Ab_t - AvgEnv) + \frac{\pi}{2} \right)$$

where Abt is the abundance of prey in the cell (Abund (X_{seal},Y_{seal})) where the female is located at time t, and AvgEnv the average abundance among non-zero cells in the environment.

When Abt << Abt-1, μ1 tends to π, the resource is lower than in the previous cell, so the female tends to come back where it was before.

When Abt >> Abt-1 or Abt = Abt-1, µ1 tends to 0, the resource seems better ahead, so the female continues its globally straight direction. The path becomes even straighter as the difference between Abt and Abt-1 increases.

When there is no difference, the female continues exactly along a straight line. This allows not to waste time in a forage-free environment. σ1 varies from 0 to 60 degrees.

When the patch is of bad quality, σ1 tends to 0 so the female goes towards µ1 with a higher probability. On the contrary when the patch is of good quality, σ1 tends to 60°, which leads to a wider exploration of the area. If females are too far from the island (further than 500 km), they change their swimming direction (SwimD) and progressively come back to the island.

When females decide either to come back to the island to feed their pups, or to go to a precise cell, other equations were used:

If $Y_{seal} > Y_{point}$ $$SwimD = \mathrm{atan}\left(\frac{X_{point} - X_{seal}}{Y_{seal} - Y_{point}}\right)\frac{180}{\pi} - 180$$

If $Y_{seal} < Y_{point}$ $$SwimD = \mathrm{atan}\left(\frac{X_{point} - X_{seal}}{Y_{seal} - Y_{point}}\right)\frac{180}{\pi}$$

If $Y_{seal} = Y_{point}$ $$SwimD = signof\,(X_{seal} - X_{point})\frac{\pi}{2}$$

where X_{point} and Y_{point} are the coordinates of either the island or the memorized best cell depending on the decision, and X_{seal}, Y_{seal} the female coordinates. To determine the position of the female given a swimming direction, we used the following equations:

$$X_{seal}(t+1) = X_{seal}(t) - step \cdot length_{step}\ \sin(SwimD\frac{\pi}{180}),$$

$$Y_{seal}(t+1) = Y_{seal}(t) + step \cdot length_{step}\ \cos(SwimD\frac{\pi}{180}),$$

where $length_{step}$ is the number of cells travelled in an hour, and step is 0 if the female is fishing or 1 if not.

D. Fishing or moving

The probability of fishing depends on various parameters. It is set to zero during the day, lantern fishes staying at great depth. During the night, if a female is not at satiation, it is possible for her to fish, depending both on prey abundance and her energetic stock (Table 2). The satiation scales allometrically with body mass and cannot exceed the maximal energy of the female which is equal to 1.07 times the mass of the animal at the end of the former night [5]. The probability of fishing is based on a few assumptions:

- A poor zone was defined as a zone where the abundance of prey is lower than the mean abundance in the non-zero environment (AvgEnv).
- The more the prey, the higher the probability for females to fish.
- We define the ratio E_{seal} / E_{min} where E_{seal} is the current content in energy of the female and E_{min} the limit energy levels below which it dies. The higher this ratio, the lower the probability of fishing in a poor area. This means that females in poor body condition will be more prone to fish on poor quality patches than others.

Finally, below a minimal abundance of 1.5 (≈ 55 g) in the cell visited, fishing is not profitable since the expense due to fishing is higher than the gain. This minimal abundance will allow the female to collect at least 4×E_{mh} (where E_{mh} is the energy expenditure per hour and the constant 4 is the conversion unit of fish mass (g) into kcal) in one hour (see Maternal energy balance section above). This energy intake rate is profitable as it compensates for both travel costs and the non-fishing hours during the day.

We defined a variable $coef_t$, which takes into account the female energy state, and includes the calculation of the variable th_t. The variable $th_t$is a threshold in prey abundance for which the probability of fishing is 0.5. We had two cases:

If $\frac{E_{seal}(t)}{E_{init}} \geq 1.0$ then $coef_t = 0$ and $th_t = AvgEnv$

If $\frac{E_{seal}(t)}{E_{init}} \leq 0.7$ then $coef_t = 1$ and $th_t = 1.5$

where E_{init} is the initial energy of the female just after it gave birth. We considered that $coef_t$ is a linear function of $\frac{E_{seal}(t)}{E_{init}}$ with coefficients a and b to be determined. We thus solved:

$$\begin{cases} 0 = a + b \\ 1 = 0.7a + b \end{cases} \Leftrightarrow \begin{cases} a = -\frac{1}{0.3} \\ b = \frac{1}{0.3} \end{cases}$$ and obtained:

$$coef_t = \frac{1}{0.3}\left(1 - \frac{E_{seal}(t)}{E_{init}}\right)$$

So we defined th_t:

$$th_t = 1.5 + (1 - coef_t) \times (AvgEnv - 1.5)$$

Finally, the probability to fish at the location *i* during the night is defined by the following sigmoid equation:

$$P(fishing)_{i,t} = \frac{e^{2(prey\ abundance_i - th_t)}}{1 + e^{2(prey\ abundance_i - th_t)}}$$

where prey $abundance_i$ is the abundance of prey at time t in the cell i and 2 is an ad hoc value to translate the curve so that P(fishing) ≈ 0 when prey abundance is nearly 0.

E. Foraging or returning

For a fixed ts, the probability to come back to the island is the Normal cumulative distribution N(μ_3, σ_3), μ_3 being a linear and decreasing function of ts. Therefore, the more the time spent at sea, the more it is inclined to return to the island. At ts = 0, the probability to return must equal 1.0 only when E = E_{max}. At ts = T_{max}, μ_3 = E_{wb}. Then, if the maximum time T_{max} is reached, even if it is only slightly profitable to their pups, females come back. If not, they stay at sea and abandon their pups to find enough food to survive. Next, the simulation stops when the pup dies from starvation. Then:

$$\mu_3 = \frac{E_{wb} - 100}{100} \times ts + 100\ .$$

For σ_3, we have to differentiate whether ts is below or above 50:

If *ts* < 50 then $\sigma_3 = (100 - \mu_3)/3$

and if *ts* > 50 then $\sigma_3 = (\mu_3 - Ewb)/3$.

Division by 3 is because approximately 98% of the values of a Normal distribution are between $\mu_3 \pm 3\sigma_3$. For every E_{wb}, we obtain a graph presented Fig A.

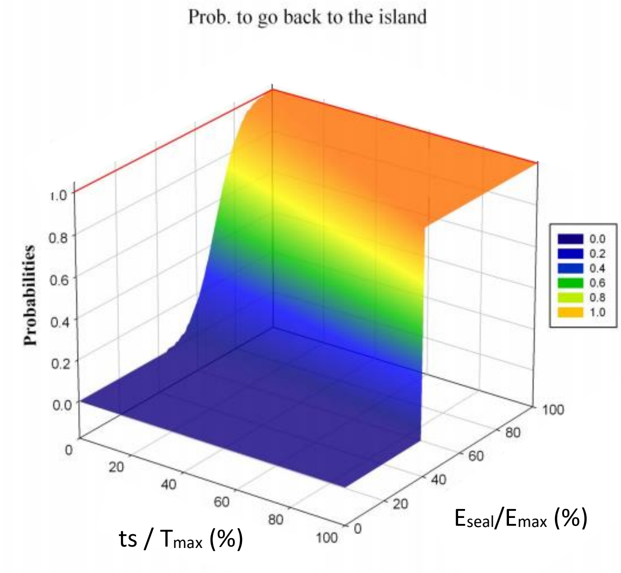

Fig A. Probability for the female to return to the island. Normal Cumulative distribution of the probabilities for the female to return to the island for a fixed amount of energy necessary to head back to the island (E_{wb}). The x axis corresponds to time ts, the y axis to the female energy divided by its maximal energy capacity.

F. Memorizing

When memorization applies, we considered that the swimming direction taken right after leaving the island follows a Normal distribution $N(\mu_2, \sigma_2)$ with μ_2 the swimming direction toward the memorized point and:

$$\sigma_2 = aS + b,$$

where S is the fishing success during the previous trip measured as the energy gained during the last trip divided by the time it took to acquire it and S < 0. If the success is very low, the seals explore more intensively, σ_2 should equals 60 and the direction will have 98% chance to fall between $\mu_2 \pm 180$. If the success is maximal, females should move almost straight to the memorized point, hence $\sigma_2 = 0$. Then, we defined S = 1.55 corresponding to a hypothetical maximal storable energy in four days, that is 149 MJ in 96 hours. We concluded that:

$$\sigma_2 = -35.5 \cdot S + 60$$

All these values are valid for any trips except for the *post-partum* one, since there has been no recording of the energy during the prenatal period.

G. Cost/benefit ratio

The search for an optimum value of (R = E/SP) was done by calculating the ratio of a second order polynomial regression on E with a Hill function fitted on SP:

$$SP\left(Length\right)=\frac{\alpha}{1+\left(\frac{\beta_1}{Length}\right)^{\beta_2}} \quad (1)$$

$$E\left(Length\right)=\alpha_1+\alpha_2 Length+\alpha_3 Length^2 \quad (2)$$

where *Length* is the seal length (cm) and α, α1,α2, α3, β1 and β2 the parameters to be estimated. A classical gradient method was then used to calculate:

Min[R(*Length*) = E(*Length*)/SP(*Length*)].

Results from simulations (SP and E) used for fitted function are in Table A and Fig 7 can be reconstructed using parameters indicated in Table B.

Table A: Average success of female pup pairs (SP) and energy spent (E) in function of each distance to the resource and the seal body length.

Distance (km)	150		200		250		300		350		400	
Seal Length (cm)	SP	E (MJ)	SP	E (MJ)	SP	E (MJ)	SP	E (MJ)	SP	E (MJ)	SP	E (MJ)
85	0.10	404.99	0.10	337.99	0.05	303.18	0.01	286.87	0.01	268.56	0.01	255.97
100	0.57	1325.66	0.51	1246.52	0.21	920.58	0.17	887.03	0.04	636.37	0.01	539.79
115	0.80	1968.56	0.83	1954.43	0.63	1698.05	0.53	1558.20	0.34	1297.88	0.17	1080.77
130	0.81	2414.68	0.89	2448.71	0.72	2165.13	0.70	2113.42	0.49	1788.41	0.33	1593.10
145	0.82	2924.81	0.87	2875.78	0.77	2654.33	0.74	2599.93	0.57	2270.91	0.44	2065.49
160	0.83	3392.09	0.88	3367.34	0.75	3012.48	0.76	3025.08	0.60	2753.57	0.42	2393.17
175	0.86	3891.94	0.87	3809.82	0.78	3542.76	0.75	3440.56	0.58	3023.46	0.50	2886.45

Table B: Estimated parameters of fitted functions (1) and (2).

Distance (km)	SP estimated parameters				E estimated parameters			
	α	β_1	β_2	Coefficient of determination	α_1	α_2	α_3	Coefficient of determination
150	0.833	95.56	16.90	0.997	-4897.509	76.288	-0.151	0.995
200	0.883	97.854	15.473	0.998	-5532.613	85.878	0.188	0.996
250	0.763	105.466	16.484	0.995	-4663.022	69.604	-0.132	0.996
300	0.754	108.6	14.909	0.999	-4526.541	66.703	-0.121	0.999
350	0.579	113.52	16.215	0.992	-3635.141	51.334	-0.074	0.995
400	0.473	121.312	13.282	0.985	-2433.659	31.323	-0.005	0.995

References

1. Arnould JPY, Boyd IL, Speakman JR, Measuring the body composition of Antarctic fur seals (*Arctocephalus gazella*), validation of hydrogen isotope dilution. Physiol Zool. 1996; 69, 93-116.
2. Goldsworthy SD, Lea MA, Guinet C, Comparison of mass-transfer and isotopic dilution methods for estimating milk intake in Antarctic fur seal pups. Polar Biol. 2004; 27, 801-809.

3. Guinet C, Lea M-A, Goldsworthy S, Mass change in Antarctic fur seal (*Arctocephalus gazella*) pups in relation to maternal characteristics at Kerguelen Islands. Can J Zool. 2000; 78, 476-483.
4. Boyd IL and McCann TS, Pre-natal investment in reproduction by female Antarctic fur seals. Behav Ecol Sociobiol. 1989; 6, 377-385
5. Winship AJ, Hunter AMJ, Rosen DAS, Trites AW, Food consumption by seal lions: exixting data and techniques. Alaska Sea Grant, University of Alaska Fairbanks. 2006; p 177-191.
