# Supplementary material for: Breeding success of a marine central place forager in the context of climate change: A modeling approach
Source: PLoS One. 2017 Mar 29;12(3):e0173797. doi: 10.1371/journal.pone.0173797 (PMC5371308; doi:10.1371/journal.pone.0173797)
Supplement: S2 Text — (PDF) [file pone.0173797.s002.pdf]

S2 Text. Details of the linear relationship between distance to the resource and female body length.

We define Dist the average distance from colony to the fishing areas and L the average length of the animals. Let us assume the speed at which the fishing areas move away from the island and the speed at which the female body length can change in the course of the successive generations are constants: k_1 and k_2 respectively. Thus we have:

$$\frac{d(Dist)}{dt} = k_1 \text{ and } \frac{d(L)}{dt} = k_2$$

Integrating these equations, it comes:

$$Dist = k_1 t + D_0 \qquad (1)$$

and

$$L = k_2 t + L_0 \qquad (2)$$

Then, since $t = (Dist - D_0) / k_1$, we obtain from (2):

$$L = \frac{k_2}{k_1}\left(Dist - D_0\right) + L_0$$

Where D_0 and L_0 are the initial values of distance and of the seal length respectively.
