# Supplementary material for: Breeding success of a marine central place forager in the context of climate change: A modeling approach
Source: PLoS One. 2017 Mar 29;12(3):e0173797. doi: 10.1371/journal.pone.0173797 (PMC5371308; doi:10.1371/journal.pone.0173797)
Supplement: S1 Fig — (PDF) [file pone.0173797.s003.pdf]

S1 Figure. Details about creation of the environmental maps

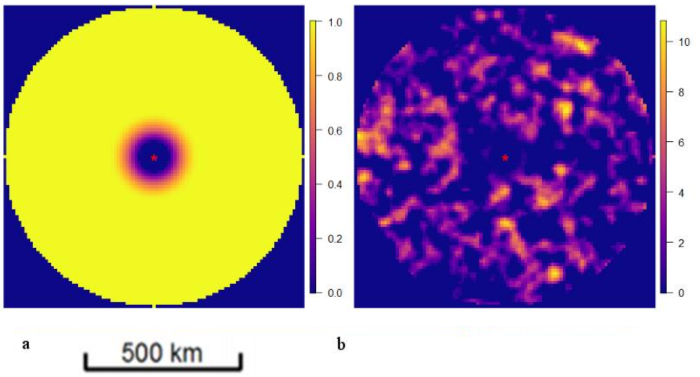

Figure A. Map creation steps.
The central red dot represents the island. (a) Example of gradient matrix with Dist = 150 km. Cells further than 500 km away from the map center, and those within the 50 km wide island plateau, are set to zero. (b) Final map: the warmer the color, the higher the abundance.

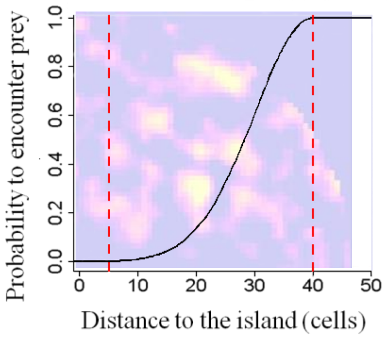

Figure B. Representation of a resource gradient around the central island.
The first vertical red line represents the edge of the island plateau (Dist = 50 km) and the second one represents the distance (Dist = 400 km) at which the probability of finding an optimal abundance is equal to 1.0. From 0 to 50 km the abundance is set to 0.0. The black curve depicts the abundance gradient from 0.0 to 1.0.

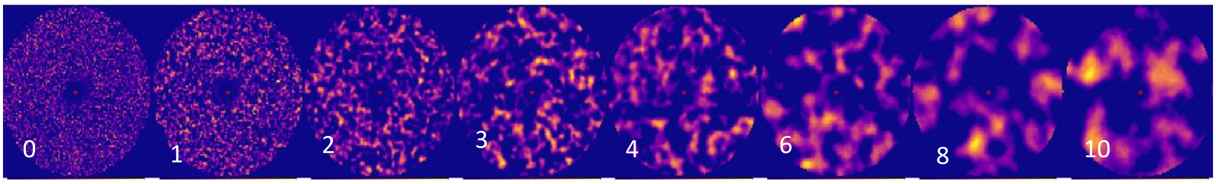

Figure C. Examples of maps with various aggregation levels.
The number represents the aggregation levels from 0 to 10. The central red dot represents the island.
