# Supplementary material for: Breeding success of a marine central place forager in the context of climate change: A modeling approach
Source: PLoS One. 2017 Mar 29;12(3):e0173797. doi: 10.1371/journal.pone.0173797 (PMC5371308; doi:10.1371/journal.pone.0173797)
Supplement: S2 Fig — (PDF) [file pone.0173797.s004.pdf]

S2 Figure. Comparison between real tracking and tested movements in the simulator.

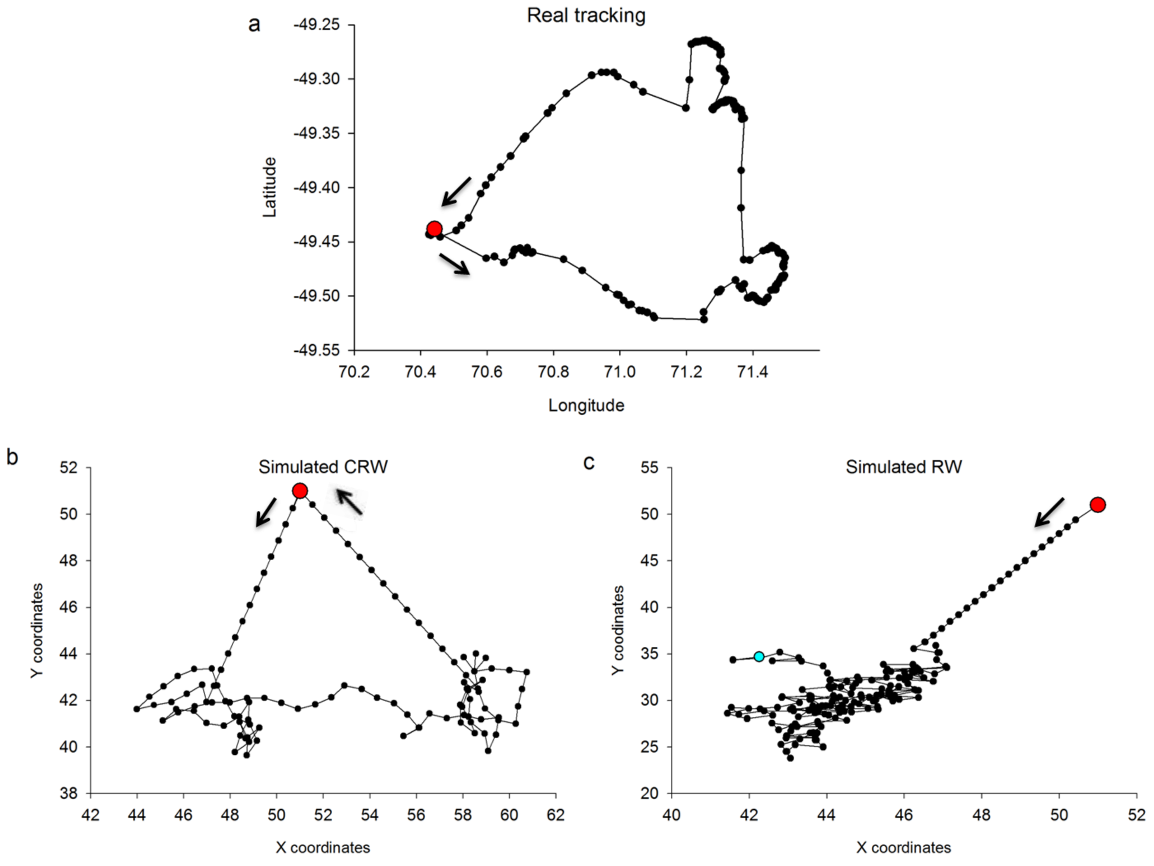

Figure A. The red point represents the island location, and arrows represent the direction taken by the female. (a) Real tracking of a female (data). (b). Simulated tracking of a female using a correlated random walk. (c). Simulated tracking of a female using a pure random walk. The blue point represents the place where the female died within the simulation of the rearing period. Simulation parameters (b and c): Dist = 150 km, female body length = 115 cm; aggregation level = 3; intermediate abundance (180 g h^{-1}); Type of memory = Mem1.
